# Population-based estimate for the correlation of the Oncotype Dx Breast Recurrence Score® result and Ki-67 IHC MIB-1 pharmDx in HR+, HER2−, node-positive early breast cancer

**DOI:** 10.1186/s13058-022-01571-7

**Published:** 2022-11-01

**Authors:** Michael Crager, Sameera R. Wijayawardana, Aaron M. Gruver, Andrea Blacklock, Christy Russell, Frederick L. Baehner, Francisco Sapunar

**Affiliations:** 1 Biostatistics – Exact Sciences Corporation, Redwood City, CA USA; 2 Global Statistical Sciences – Oncology, Eli Lilly and Company, Indianapolis, IN USA; 3 Clinical Diagnostics Laboratory, Eli Lilly and Company, Indianapolis, IN USA; 4 Medical – Exact Sciences, Redwood City, CA USA; 5 Medical Affairs – Exact Sciences, Redwood City, CA USA; 6 Global Medical Affairs, Eli Lilly and Company, Basingstoke, UK; 7 Lilly Corporate Center, Indianapolis, IN 46285 USA

**Keywords:** Early breast cancer, Immunohistochemistry, Ki-67, MIB-1, Oncotype Dx, Recurrence score

## Abstract

**Background:**

The United States Food and Drug Administration recently approved a Ki-67 immunohistochemistry (IHC) assay to identify patients with early breast cancer at high disease recurrence risk. The Oncotype Dx Breast Recurrence Score® assay has been validated in hormone receptor-positive (HR+), human epidermal growth factor receptor 2-negative (HER2−) invasive breast cancer (IBC) to predict chemotherapy benefit and distant recurrence risk, regardless of nodal status. This study assessed the correlation between Recurrence Score® (RS) results and the Ki-67 IHC MIB-1 pharmDx assay.

**Methods:**

HR+, HER2−, N1 IBC samples with RS results were examined by Ki-67 IHC; 311 specimens were collected, including 275 without regard to RS (“unselected RS”) and 36 more with RS 26–100; 12 were lymph node negative upon pathology report review, and one had no Ki-67 score, leaving 262 unselected RS and 298 total samples. Spearman rank correlation was calculated using the unselected samples and a weighted rank correlation using all samples. A receiver operating characteristic (ROC) curve for predicting high RS (26–100) from Ki-67 was constructed.

**Results:**

The Spearman rank correlation between Ki-67 and RS results was moderately positive (unselected RS samples: 0.396; 95% confidence interval [CI] 0.288–0.493; all samples: 0.394; 95% CI 0.294–0.486). While 71% of samples with RS 26–100 had Ki-67 ≥ 20%, 75% with RS 0–25 had Ki-67 < 20%. ROC area under the curve was 0.792 (95% CI 0.725–0.859).

**Conclusions:**

The moderately positive correlation is consistent with previous analyses suggesting the Oncotype Dx® assay and Ki-67 IHC MIB-1 assay should not be used interchangeably in clinical practice.

## Introduction

Recently, an investigational Ki-67 immunohistochemistry (IHC) assay from the monarchE trial was commercially approved by the United States (US) Food and Drug Administration (FDA; approval date: October 12, 2021) to aid in identifying patients with early breast cancer (EBC) at high disease recurrence risk [1], for whom adjuvant abemaciclib in combination with endocrine therapy treatment was considered, based on data from the monarchE trial in adult patients with hormone receptor-positive (HR+), human epidermal growth factor receptor 2-negative (HER2−), node-positive EBC with high recurrence risk [2]. Lack of standardized procedures or accepted cutoff definitions have historically limited Ki-67 IHC clinical use in some geographies [3]; however, given recent approvals, an FDA-approved companion diagnostic was proposed to ameliorate these challenges in the USA [4].

The Oncotype Dx Breast Recurrence Score® test produces a Recurrence Score® (RS) result based on the quantitative expression level of 21 genes in ribonucleic acid extracted from formalin-fixed, paraffin-embedded (FFPE) breast tumor tissue. This assay has been validated in HR+, HER2−, invasive breast cancer (IBC) to predict chemotherapy benefit and distant recurrence risk, regardless of nodal status [5–11]. In practice, the vast majority of N + patients referred for RS testing have N1 disease. For patients with HR + EBC, the RS result has been recommended to guide chemotherapy treatment decisions [12–15].

Prior studies have reported a moderate association between Ki-67 and RS result [16–18], but the relationship has not been evaluated using the Ki-67 IHC MIB-1 pharmDx assay. Gaining a better understanding of how genomic risk assessments relate to IHC markers will continue to improve clinical decision making for patients with HR+, HER2− EBC. The objectives of this study were to estimate the rank correlation between the Oncotype Dx Breast RS result and the Ki-67 IHC MIB-1 pharmDx assay in HR+, HER2−, node-positive (1–3 positive nodes; N1) EBC, and to estimate the proportion of samples with Ki-67 IHC ≥ 20% among those with RS > 25 or RS > 30.

## Methods

Deidentified human tumor tissue was obtained according to protocols and procedures approved by the Western Institutional Review Board (WIRB)-Copernicus Group (WCG) Institutional Review Board (IRB; 01-261). This study was conducted in accordance with the Declaration of Helsinki. A minimum of 275 and a maximum of 355 consecutive EBC tumor sample specimens meeting inclusion criteria were anticipated from Exact Sciences (Redwood City, CA). Samples from US patients with HR+, HER2−, N1 IBC with an available RS result and sufficient tumor content were eligible. N1 status was initially based on physician assessment and confirmed by central review of pathology reports. Eligible samples had to have FFPE blocks with ≥ 1.1 cm longest linear length of tumor assessed on the hematoxylin and eosin slide with ≥ 200 invasive tumor cells. RS result was not considered during acquisition of the first 275 samples. After 275 samples were acquired, if the proportion of specimens with RS 26–100 was < 29%, additional consecutive specimens with RS 26–100 were to be acquired to obtain 80 total samples with RS 26–100.

For each IBC FFPE specimen, four unstained tissue sections of 5-micron-thick were prepared by trained histotechnologists, as per the instructions for use, and sent to Agilent Technologies (Santa Clara, CA) for Ki-67 IHC assessment using the Ki-67 IHC MIB-1 pharmDx assay [1]. Two trained and certified pathologists assigned a percentage of Ki-67 positivity and a Ki-67 diagnostic category (positive/negative) to each specimen using a ≥ 20% cutoff, as per scoring instructions developed for monarchE. For specimens with discordant scores (prespecified as either a discordant diagnostic category or a positivity percentage varying by > 10 points for blocks with a mean score < 30% and by > 25 points for blocks with a mean score ≥ 30%), a third trained and certified pathologist performed a blinded review. The consensus diagnostic category (positive/negative) and an average of the two closest positivity percentage scores were prespecified as the final dataset. All Ki-67 results were produced without knowledge of the associated RS.

### Statistical analysis

The primary Spearman rank correlation assessment between RS result and Ki-67 percent positivity used the initial acquisition, when all RS results were accepted (*Unselected RS*), to have a study population representative of patients with samples sent for RS testing. Additionally, a weighted Spearman rank correlation was calculated using all samples, upweighting samples with RS 0–25 to create a virtual population having the same overall RS result distribution as the unselected population. Cross-tabulations of the Ki-67 diagnostic category (< 20% vs ≥ 20%) versus RS result (0–25 vs 26–100; and 31–100) were made with all samples. An exploratory analysis examined the true positive and false positive rates for prediction of RS 26–100 by Ki-67 using a receiver operating characteristic (ROC) curve.

## Results

**Fig. 1:**
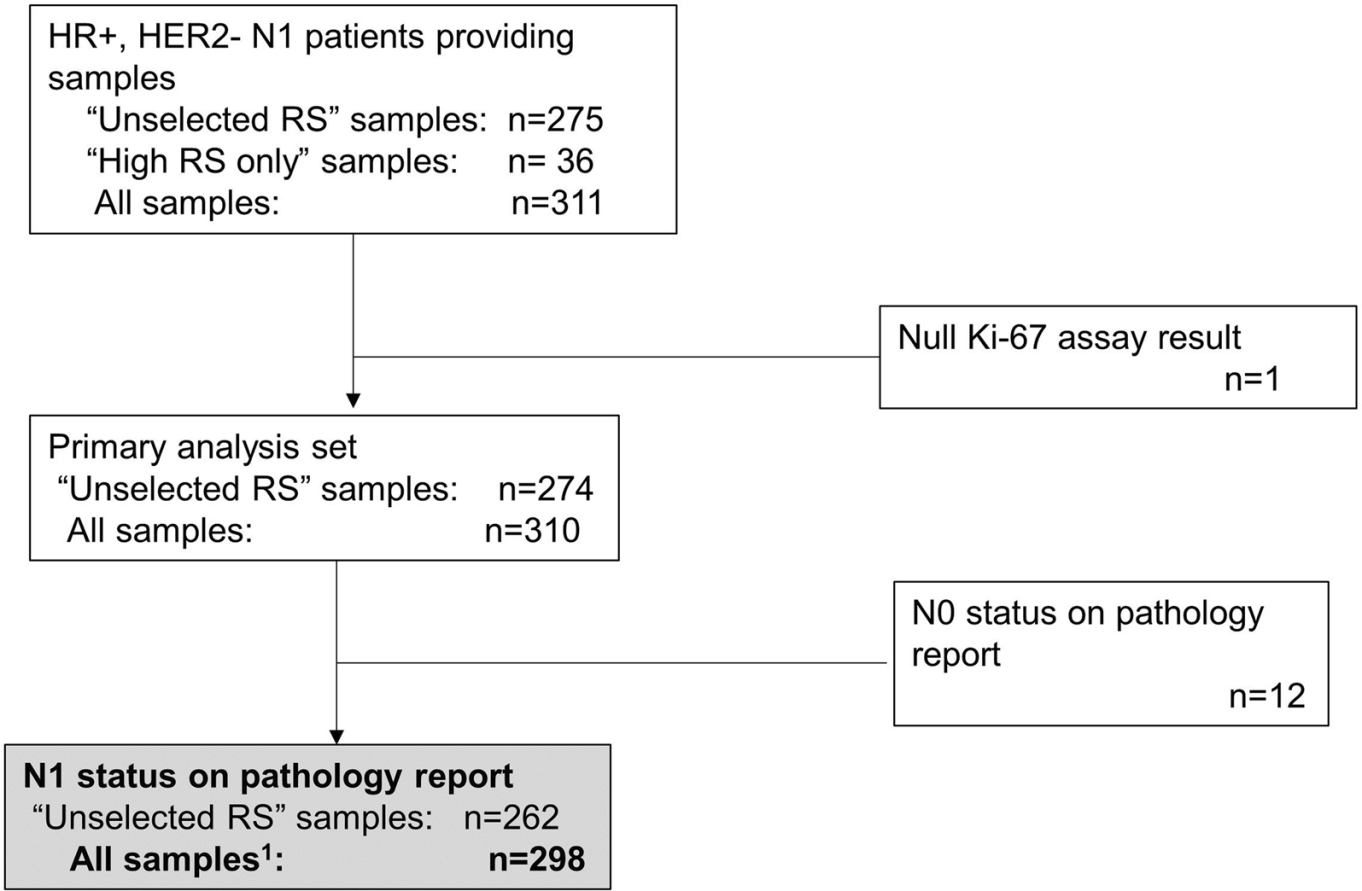
Derivation of analysis dataset (n = 298). ^1^Includes 80 samples with RS 26–100. HER2−, human epidermal growth factor receptor 2-negative; HR+, hormone receptor-positive; n, sample size; N0, node-negative; N1, 1–3 positive lymph nodes; RS, Recurrence Score®

A total of 311 samples were collected, of which one had a null Ki-67 result. Examination of pathology reports further excluded 12 node-negative specimens, leaving 262 (*Unselected RS*) and 298 (*All Samples*) samples (Fig. 1). Three patients with N2 or N3 disease were included in the analysis. Patient demographic and disease characteristics are given in Table 1.

**Table 1:** Patient demographic and disease characteristics

Characteristic	Count (%) in all samples n = 310	Count (%) excluding N0 samples n = 298
*Age category*		
≤ 50 years	51 (16)	48 (16)
> 50 years	259 (84)	250 (84)
*Sample type*		
Non-Biopsy	270 (87)	258 (87)
Biopsy	40 (13)	40 (13)
*T-stage*^*1*^		
T1	2 (1)	2 (1)
T1a	3 (1)	3 (1)
T1b	2 (1)	1 (< 1)
T1c	97 (37)	94 (37)
T2	142 (54)	135 (54)
T3	15 (6)	14 (6)
T4	3 (1)	3 (1)
Unknown^2^	46	46
*N-stage*^*1*^		
N0	12 (5)	0
N1	227 (87)	227 (92)
N1mic	18 (7)	18 (7)
N2	2 (1)	2 (1)
N3	1 (< 1)	1 (< 1)
Unknown^2^	50	50
*AJCC anatomic stage*^*1*^		
IA	4 (2)	0
IIA	104 (40)	97 (39)
IIB	133 (51)	132 (53)
IIIA	16 (6)	16 (6)
IIIB	2 (1)	2 (1)
IIIC	1 (< 1)	1 (< 1)
Unknown^2^	50	50

^1^Denominator for calculation of percentages in T-stage, N-stage, and AJCC anatomic stage categories did not include those samples with unknown stage

^2^Stage could be unknown due to missing pathology report (n = 6), unclear report entry (n = 4), or because sample was from a biopsy (n = 40)

**Fig. 2:**
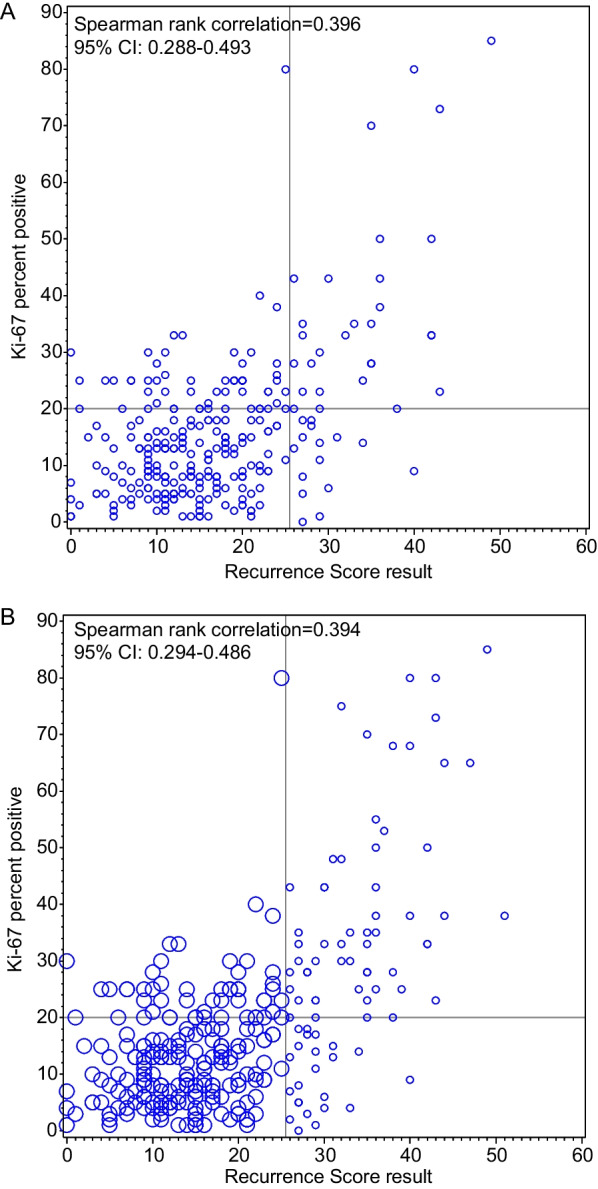
Scatter plot of percentage Ki-67 positivity with RS result for *Unselected RS* pool (n = 262; **A**) and for *All Samples* (n = 298; **B**). Circle diameters in **B** are reflective of respective weights in weighted analysis. n, sample size; RS, Recurrence Score® result

**Fig. 3:**
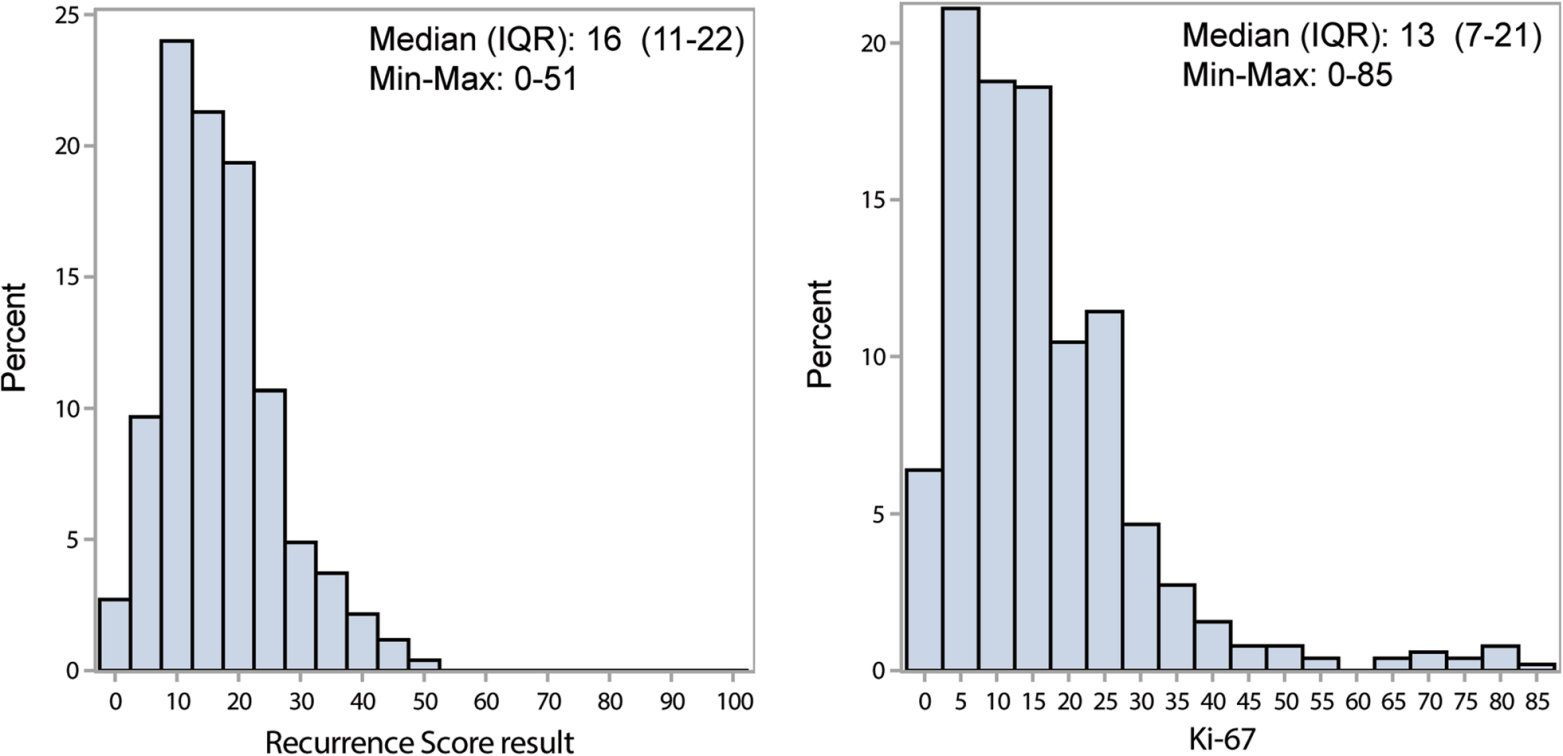
Distributions of Recurrence Score® results and percentage Ki-67 positivity among all samples with N1 status confirmed (n = 298 using weighting). IQR, interquartile range; max, maximum; min, minimum; n, sample size; N1, 1–3 positive lymph nodes

The Spearman rank correlation between Ki-67 and RS result in the *Unselected RS* samples was moderately positive (0.396; 95% confidence interval [CI] 0.288–0.493; Fig. 2a). The proportion of samples with RS 26–100 among the *Unselected RS* sample and when screening 308 additional N1, HR+, HER2− samples to obtain 47 samples with high RS result (36 of which met the study entry criteria) was 15.6%. There were 80 samples with RS 26–100, so the overall weighted virtual sample size was 80/0.156 = 511.6, with 511.6–80 = 431.6 samples having RS 0–25. Accordingly, the 298–80 = 218 collected N1 samples with RS 0–25 were upweighted in the virtual population by a factor of 431.6/218 = 1.980. The weighted Spearman rank correlation using *All Samples* was similar to the primary result (0.394; 95% CI 0.294–0.486; Fig. 2b). Among *All Samples* with RS 0–25, 164 (75%) were Ki-67 < 20% and 54 (25%) were Ki-67 ≥ 20%. Among *All Samples* with RS 26–100, 23 (29%) were Ki-67 < 20% and 57 (71%) were Ki-67 ≥ 20%. Among samples with RS 31–100, 5 (11%) were Ki-67 < 20% and 40 (89%) were Ki-67 ≥ 20% (Table 2; Fig. 3).

**Table 2:** Distribution of Ki-67 diagnostic category and RS result among all samples with N1 status confirmed (n = 298)

RS group, n (%)	All samples, N1 (n = 298)		
	Ki-67 diagnostic category		
	Negative	Positive	Total
0–25	164 (75)	54 (25)	218
26–100	23 (29)	57 (71)	80
Total	187 (63)	111 (37)	298
31–100^1^	5 (11)	40 (89)	N/A

n, sample size; N/A, not applicable, as this is a subset of the 26–100 RS group, N1, 1–3 Positive lymph nodes; RS, Recurrence Score®

^1^A subset of RS group 26–100

**Fig. 4:**
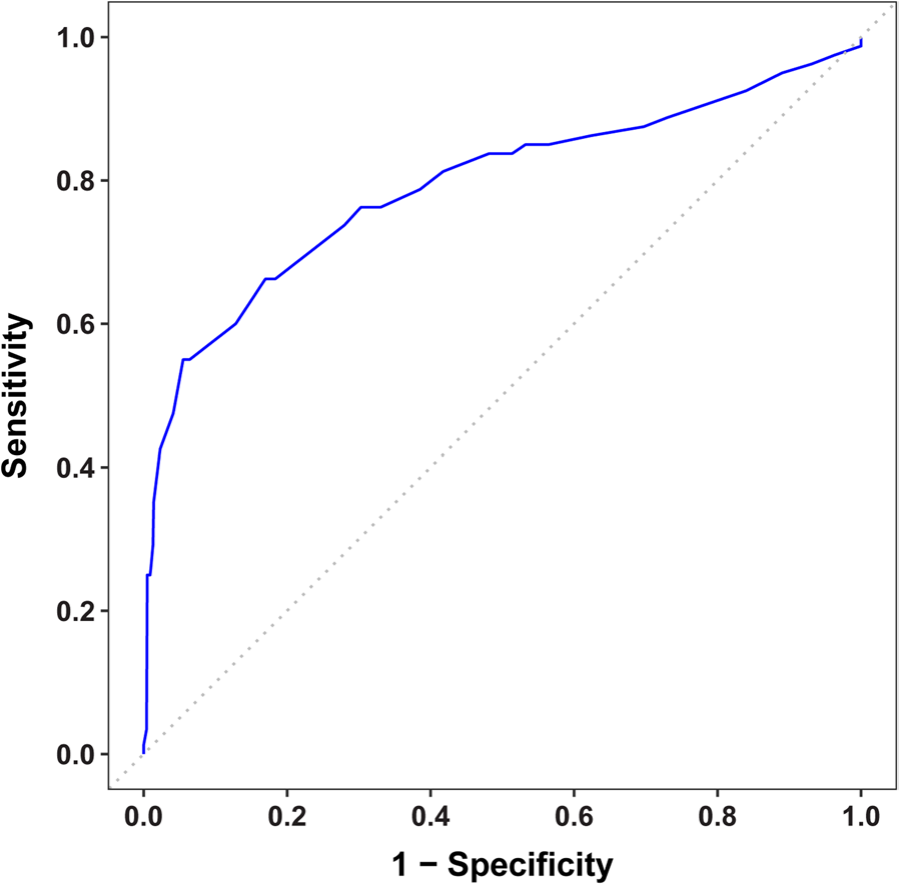
Receiver operating characteristic curve showing Ki-67 as a predictor of Recurrence Score® result for all samples with confirmed N1 status (n = 298). n, sample size; N1, 1–3 positive lymph nodes

An exploratory ROC curve analysis found an area under the curve of 0.792 (95% CI, 0.725–0.859) (Fig. 4). Weighting was not necessary when computing the ROC curve using *All Samples*, as the same weights would be applied to the numerator and denominator when calculating the true positive and false positive rates.

## Discussion

Ki-67 expression has been widely studied as a cell proliferation marker and is an independent prognostic factor in EBC [19]. These assays suffered from lack of analytical validity (preanalytical, analytical, and interpretation and scoring differences across laboratories), limiting widespread implementation of Ki-67 as a diagnostic assay in the USA [3, 20]. Ki-67 IHC MIB-1 pharmDx analytical validation studies have shown high concordance and reproducibility across laboratories and instruments when using an optimized protocol and standardized qualitative scoring approach [1]. The assay was used and clinically validated as a prognostic biomarker in the phase 3 monarchE trial. Given the clinical utility of both assays in HR + , HER2− node-positive EBC treatment decision making, we investigated the correlation between the RS result and the Ki-67 IHC assay, in HR + , HER2−, N1 EBC sent for RS central laboratory testing. In this study, the correlation between RS result and Ki-67 expression in EBC was moderately positive in the unselected and the overall enriched RS samples. The intention of testing the correlation in both samples was to include a representative proportion of patients with RS 26–100, given the known distribution of the RS result in the general population [21].

In this first study reporting the correlation between RS result and a standardized, FDA-approved test for Ki-67 IHC in lymph node positive patients, results generally aligned with prior studies showing a moderately positive association between other non-standardized Ki-67 IHC assays and RS results [16–18]. The first central assessment of Ki-67 and the RS result was a prospective, randomized, multicenter, phase 3, chemotherapy trial in HR+, HER2− EBC in 3198 patients, where the Spearman correlation was weak to moderately positive (quantitative or semi-quantitatively assessed, both < 0.4) [16].

Among samples with RS 26–100 in the enriched, *All samples* set, 71% had high Ki-67, suggesting a stronger relationship between Ki-67 and the RS result within that subgroup. This may be due to the association between Ki-67 and the Onctoype DX test’s proliferation module, which is more influential in high RS results [7].

Results should be considered within the context of study strengths and limitations. The dataset represented a series of samples prospectively collected under a predefined protocol. Clinical outcomes were not available in the dataset. The Ki-67 IHC MIB-1 pharmDx was approved as a qualitative assay based upon the predefined cutoff used in the monarchE trial rather than the continuous scores used in some exploratory analyses presented. Strengths include a prespecified study analysis plan, central assay testing, and two trained pathologists doing all IHC assessments in parallel with a third pathologist performing blinded assessments in case of significant discordances.

## Conclusion

This study added to existing knowledge by showing a moderate association between the Oncotype Dx Breast Recurrence Score® result and the standardized Ki-67 IHC MIB-1 pharmDx assay and elucidated the relationship between a high RS result (26–100) and Ki-67 IHC. The correlation was similar to those observed in earlier studies using other Ki-67 assays. Data presented here, aligned with previous analyses, suggest that the Oncotype Dx assay and Ki-67 IHC MIB-1 assay should not be used interchangeably in clinical practice.

## Data Availability

The study data may be obtained upon reasonable request and agreement from both Eli Lilly and Company and Genomic Health, Inc., a wholly owned subsidiary of Exact Sciences Corporation.

## Abbreviations

CI: Confidence interval
EBC: Early breast cancer
FDA: Food and Drug Administration
FFPE: Formalin-fixed, paraffin-embedded
HER2−: Human epidermal growth factor receptor 2-negative
HR+: Hormone receptor-positive
IBC: Invasive breast cancer
IHC: Immunohistochemistry
IRB: Institutional Review Board
N1: 1–3 Positive lymph nodes
ROC: Receiver operating characteristic
RS: Recurrence Score® result
US: United States
